# Comparative Study of the Chemical Compositions and Antioxidant Activities of Fresh Juices from Romanian Cucurbitaceae Varieties

**DOI:** 10.3390/molecules25225468

**Published:** 2020-11-23

**Authors:** Anna Cazanevscaia Busuioc, Andreea-Veronica Dediu Botezatu, Bianca Furdui, Costel Vinatoru, Filippo Maggi, Giovanni Caprioli, Rodica-Mihaela Dinica

**Affiliations:** 1 Department of Chemistry, Physics and Environment, “Dunărea de Jos” University of Galati, 111 Domnească Street, 800201 Galati, Romania; anna.cazanevscaia@ugal.ro (A.C.B.); veronica.dediu@ugal.ro (A.-V.D.B.); 2 Buzău Vegetable Research and Development Station, 23, Mesteacănului Street, 120024 Buzau, Romania; costel_vinatoru@yahoo.com; 3 School of Pharmacy, University of Camerino, Via S. Agostino 1, 62032 Camerino, Italy; filippo.maggi@unicam.it (F.M.); giovanni.caprioli@unicam.it (G.C.)

**Keywords:** Cucurbitaceae, HPLC, phenolic content, antioxidant capacity, iron chelating ability

## Abstract

Cucurbitaceae is a family of health-promoting plants due to their compounds with beneficial effects. The aim of this study was to analyze, for the first time, the chemical composition, the antioxidant activity and the metal chelating properties of fruit juices obtained from four different species of the Cucurbitaceae family cultivated in Romania, namely *Momordica charantia*, *Cucumis metuliferus*, *Benincasa hispida* and *Trichosanthes cucumerina*. The samples of juice were analyzed by high-performance liquid chromatography (HPLC) and all the four species displayed high levels of the two triterpenes, oleanolic and ursolic acids, and also in phenolic compounds, including catechin, (−)-epicatechin and gallic acid. The juices demonstrated significant antioxidant activity against the free radical 2,2-diphenyl-1-picrylhydrazyl (ranging from 20 to 95%,), a good iron binding ability (ranging from 7.45 ± 0.28% to 86.95 ± 0.97%) and also promising antioxidant potential against the ABTS radical (ranging from 4.97 to 32.60 μETx/mL juice). Our findings raise interesting questions for further research on Cucurbitaceae fruit juices and, consequently, their very good antioxidant potential suggests these fruits should be further explored for their protective effect against oxidative damage. This is the first time the chemical composition and antioxidant activities of fruit juices from these four Romanian Cucurbitaceae varieties have been investigated.

## 1. Introduction

In recent decades, the discovery of new safe and effective plant-derived medicines has been a major concern for researchers [1]. Many degenerative diseases, such as cardiovascular, cancer, and arthritis, occur due to metabolic by-products such as free radicals—reactive oxygen species. The interaction between antioxidants has been increasingly studied due to its antioxidative potential for increased health benefits as well as food stability [2]. Most vegetables from the Cucurbitaceae family have a rich chemical composition, which improves food health and stability because of their antioxidant power. The cucurbita–corn–bean complex formed the nutritional basis for pre-Columbian civilizations in the Western Hemisphere. One of the first species to be domesticated in the New World was *Cucurbits pepo* L. cultivation by the inhabitants of Guila Naquitz cave between 10,000 to 8000 before present (BP), preceding corn and beans by more than 4000 years [3]. The whole Cucurbitaceae familyhas many health benefits but each plant, depending on the cultivating area, has its own “baggage” of properties and influences human health in its own way. The Cucurbitaceae family has made a significant economic contribution and is used not only in many food products such as important dietary fibers sources, but also in other branches of industry such as cosmetics. Cucumber extracts, known for their cooling, healing and soothing properties, are often added to skin care products. Moreover, *Luffa aegyptiaca* Mill. mature fruits have been known from the oldest times as natural sponges [4], which provide a lot of healthy properties and help insulinemic response and changes in the intestinal function. The healthy properties of cucurbits make them helpful for decreasing cholesterol, insulin response and prevention of constipation [5]. The chemical composition of the Cucurbitaceae family includes phytochemicals with no nutritional value that occur naturally in plants. Some examples of compounds identified in these plants are tannins, carbohydrates, saponins, polyphenols and cardiac glycosides [6]. They also contain a high level of other bioactive compounds, such as triterpenes, sterols, alkaloids and carotenoids. Cucurbits display antioxidant effects thanks to their various bioactive compounds with a free radical scavenging ability, such as cucurbitacins B and E, ellagitannins or carotenoids such as α-carotene, β-carotene, lutein and zeaxanthin [6,7,8].

*Momordica charantia* L. (bitter melon) is one of the most well known and studied species of Cucurbitaceae and due to its ample and unique chemical composition, it is a very good candidate for the development of anti-obesity, anti-inflammatory and anti-neoplastic health products and medicines. Fresh juice from *M. charantia* could control blood sugar and insulin levels, as well as improving the human digestive system. The daily dose of a *M. charantia* juice drink helps to increase body stamina and prevents fatigue [9]. Bitter melon chemical composition is high in phenolic compounds, including gallic acid, tannic acid, catechin, caffeic acid, *p*-coumaric acid, ferulic acid, and benzoic acid [10,11]. Various parts of *M. charantia* have shown significantly different antioxidant activities when ferric reducing power assay were used. The antioxidant activity of unripe *M. charantia* fruit was found to be higher than that of ripened fruit, but lower than that of *M. charantia* stem and leaves [12]. Other studies revealed that non-polar extracts from this plant showed good antioxidant activity which might be attributed to the content of fatty acids and essential oils rather than phenolic-based compounds [13]. *M. charantia* has been shown to have anti-*Helicobacter pylori* activity, which is beneficial for anti-ulcer activity [14]. Two in vitro studies have highlighted the anti-herpes, ribosome-inactivating proteins MAP30 against HSV-2 and HSV-1 activities of its extracts [7].

*Cucumis metuliferus* E.Mey. ex Naudin or Kiwano is a monoecious, climbing, annual herb that can be grown practically anywhere, provided the season is warm [15]. The vegetables and leaves of this plant are widely consumed as a source with a high nutritional value and therapeutical activities. For example, oral treatment of experimental rats with *C. metuliferus* fruit extract caused a significant reduction in the blood glucose level of alloxan-induced hyperglycemic rats, although there was no significant effect on the normo-glycemic rats. [16]. The total polyphenol content in seeds of *C. metuliferus* was higher than in other parts of the plant and the peels showed a strong ferrous ion-chelating capacity, although they did not have a high polyphenol content, DPPH, or ABTS radical scavenging activities [17]. In addition to being very rich in water, the pulp has a high concentration of mineral salts, including potassium salts, and is low in sodium salts. Moreover, two important compounds have been identified in Kiwano, rutin and lutein, with pharmacologically activities which are antioxidant, anti-inflammatory, spasmolytic, capillary protective, and blood platelet aggregation inhibitory [18].

*Benincasa hispida* (Thunb.) Cogn., commonly known as Kundur (Malay) or under names such as winter melon, ash pumpkin, and white gourd, is a vegetable best suited to the medium of dry areas of the lowland tropics. It has been found that excessive humidity and prolonged overcast have unfavorable effects on the plant’s health and productivity [19,20]. Plants belonging to the genus *Benincasa* have been used as therapeutic agents for the treatment of diabetes mellitus, diuresis diseases, urinary infection, chronic inflammatory disorders [21], epilepsy, peptic ulcer and internal hemorrhages [22]. Studies on the juice of *B. hispida* revealed its stability over time and its high concentration of nutritional compounds, low sugar and low sulfides content [23]. The in vitro antioxidant activity of *B. hispida* fruit revealed that its seeds could be good sources of antioxidants, due to their high capacity for the inhibition of linoleic acid oxidation [24]. This fruit can reduce the gross intake of food and it can therefore be useful as one of the potential anti-obesity agents [25]. α- and β-Amyrins and derived acids such as oleanolic acid, ursolic acid, lupeol, betulin, betulinic acid and stigmasterol and its analogues found in *B. hispida* waxy coatings of leaves and fruits have revealed very good therapeutic activities, including antiviral, anti-inflammatory and antitumor activities [26].

*Trichosanthes cucumerina* L. (Snake cucumber) is an annual dioecious climber. It is widely distributed in Asian countries like Sri Lanka, India, Malay Penisula and Philippines [27]. *T. cucumerina* is a good source of bioactive compounds with a high antioxidant power, such as carotenoids, ascorbic acid, flavonoids, and phenolic acids [28]. *T. cucumerina* have shown significant hypoglycemic and antihyperglycemic activities, with fruits and leaves rich in polyphenolic compounds having been reported to be useful in treating liver disorders and diabetes [29]. This plant can enhance gastric mucus secretion while reducing the acidity of the gastric juice [30].

**Figure 1:**
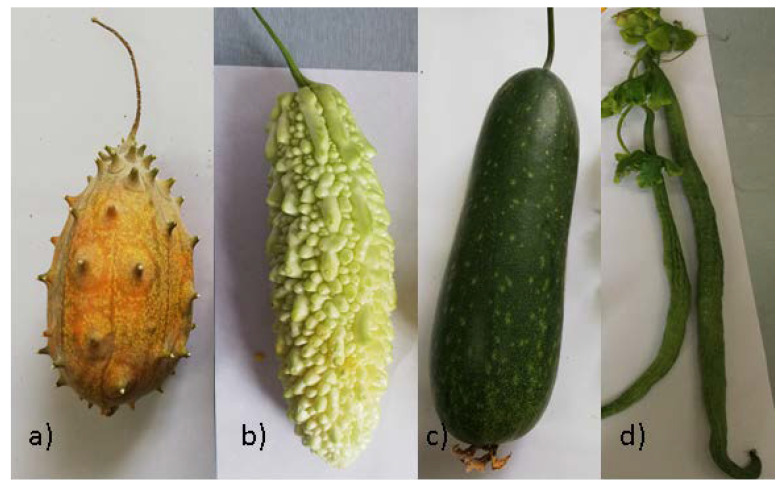
Romanian plant varieties: (**a**) *Cucumis metuliferus* “Tempus” variety, (**b**) *Momordica charantia* “Brâncuși” variety, (**c**) *Benincasa hispida* “Zefir” variety and (**d**) *Trichosanthes cucumerina* “Felix” variety.

The aim of our study was to investigate the chemical composition of fresh juices of the four mentioned species, namely *Momordica charantia* ”Brâncuși” variety, *Cucumis metuliferus* ”Tempus” variety, *Benincasa hispida* ”Zefir” variety and *Trichosanthes Cucumerina* ”Felix” variety, from the Cucurbitaceae family, acclimatized in Buzau, Romania (Figure 1). Another purpose of this study was to investigate, in a comparative way, the in vitro antioxidant activities and iron binding abilities of both individual and combined juice assortments from these plant varieties in order to stimulate the increase of consumption of fresh juices useful for a rich, diverse and health-promoting diet. The fact that scientific publications focusing on the profile and content of antioxidant compounds in fresh juices obtained from these plant species are rare, has motivated us to discover more information on the antioxidant properties of combined fruit juices that can work in a synergistic manner. The present study also highlights the potential of fresh juices as a promising and attractive source of valuable phytochemicals and natural antioxidants for the development of health products and medicines.

## 2. Results and Discussion

### 2.1. HPLC Analysis of Juices Constituents

In the present work, a simultaneous analysis of seventeen compounds, namely gallic acid, neochlorogenic acid, caffeic acid, *p*-coumaric acid, *trans*-ferulic acid, (+)-catechin hydrate, (−)-epicatechin, procyanidin A2, procyanidin B2, rutin, quercetin-3-d-galactoside, kaempferol-3-glucoside, kaempferol, quercetin, ursolic and oleanolic acids, in four different species of the Cucurbitaceae family cultivated in Romania, namely *Momordica charantia, Cucumis metuliferus, Benincasa hispida* and *Trichosanthes cucumerina* was achieved by HPLC-DAD. The quantitative data (mg kg^−1^) of the analysed compounds are reported in Table 1.

The vast majority of species in the Cucurbitaceae family contain a large number of triterpenes and significant amounts of polyphenols and flavonoids. Upon reviewing the literature data, we did not find studies about all the species analyzed in this paper. All the juices from our studied species contain significant amounts of triterpenes and flavonoids, except *B. hispida,* in which a lack kampferol and kampferol-3-glucosides has been observed. The richest chemical composition was found in the juice of *M. charantia* and *C. metuliferus*.

We performed an ANOVA analysis to detect the differences between the averages of the concentrations of the compounds from each of the four groups (*M. charantia, C. metuliferus, B. hispida* and *T. cucumerina*). The Levene test confirms the homogeneity of the variants (*p* > 0.05). Since, in the ANOVA results, *p* < 0.05 in all cases, the averages in the content of analysed compounds are significantly different for at least two of the four groups analysed. The results of the Duncan test are shown in Table 1 (if the mean values per line differ significantly between groups, then the letters labelling them are different).

The major constituents in *Momordica caranthia* were epicatechin (555.22 mg kg^−1^), catechin (201.78 mg kg^−1^) and the two triterpenes oleanolic acid (238.10 mg kg^−1^) and ursolic acid (195.44 mg kg^−1^). The main metabolite of *Cucumis metuliferus* was catechin (928.74 mg kg^−1^), while *Benincasa hispida* was rich in gallic acid (525.24 mg kg^−1^) which is the main constituent.

Oleanolic acid and ursolic acid are also abundant in *Cucumis metuliferus* (347.67 and 191.44 mg kg^−1^ respectively), *Benincasa hispida* (326.16 and 370.44 mg kg^−1^ respectively) and in *Trichosanthes cucumerina* (352.79 and 243.78 mg kg^−1^ respectively).

Other secondary metabolites occurring at noteworthy levels in *Momordica charantia* were kaempferol-3-glucoside (80.86 mg kg^−1^), procianidin B2 (38.48 mg kg^−1^) and rutin (29.79 mg kg^−1^), in *Cucumis metuliferus* rutin (33.78 mg kg^−1^) and kaempferol-3-glucoside (19.61 mg kg^−1^), in *Benincasa hispida* rutin (12.87 mg kg^−1^) and trans-ferulic acid (6.6 mg kg^−1^), in *Trichosanthes cucumerina* gallic acid (21.59 mg kg^−1^), kaempferol-3-glucoside (19.49 mg kg^−1^), and rutin (19.03 mg kg^−1^).

(−)-Catechin and (−)-epicatechin are two flavonoid stereoisomers which present a high antioxidant activity in living systems [31]. *M. charantia* and *C. metuliferus* have a higher amount than other juices. Animal studies have shown that the consumption of flavonoids from *M. charantia* is inversely proportional to the hyperglycaemia and hyperlipidaemia, a beneficial effect of the doses analysed without adverse reactions [32]. The antidiabetic properties exhibited by *M. charantia* have been linked to a number of compounds, including oleanolic acid, monodesmoside, charantin, momordin 1c [33]. In other studies about *C. metuliferus*, the flavonoids rutin, miricetin and quercetin have been identified. The methanol extract of *C. metuliferus* peel has pharmacological properties, such as antioxidant, anti-inflammatory, spasmolytic and antiviral activities [34]. Gallic acids was found to also be a major compound in *B. hispida* which is linked with its ability to reduce Type II diabetes [35]. Phenolic compounds have been reported to exhibit antidiabetic activity [36]; therefore, we consider that the antioxidant compounds present in the extracts may also play a major role in mediating the antidiabetic effects of *T. cucumerina.*

Ursolic acid, which is present in all the analyzed juices, is known for its activity against HIV-1 protease, which is a homodimeric enzyme. A beta-sheet formed of the four terminal segments provides the main driving force for dimerization of the per se inactive protomers [37].

### 2.2. Total Polyphenol Content

**Figure 2:**
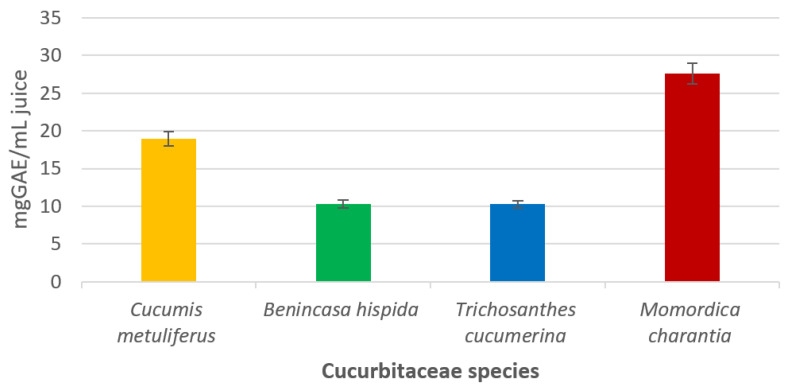
Total polyphenol content of fresh juices. The error bars represent ±standard deviation of three replicates.

Figure 2 shows the total phenolics content (TPC) of the tested samples, determined by the Folin–Ciocalteu colorimetric method. The TPC of *C. metuliferus* was found at a good concentration, expressed as 18.97 ± 1.25 mg GAE/mL sample. In comparison, the content of TPC was found in smaller amounts of 10.32 ± 1.06 mg GAE/mL in *B. hispida* and 10.253 ± 0.56 mg GAE/mL in *T. cucumerina* samples. Moreover, a higher level of TPC expressed as 27.64 ± 1.89 mg GAE/mL sample was found in the *M. charantia* juice variety. These results may be due to phenolic compounds such as gallic acid, 3-*O*-caffeoylquinic acid, 5-*O*-caffeoylquinic acid, caffeic acid, *p*-coumaric acid and *trans*-ferulic acid.

### 2.3. Total Flavonoids Determination

**Figure 3:**
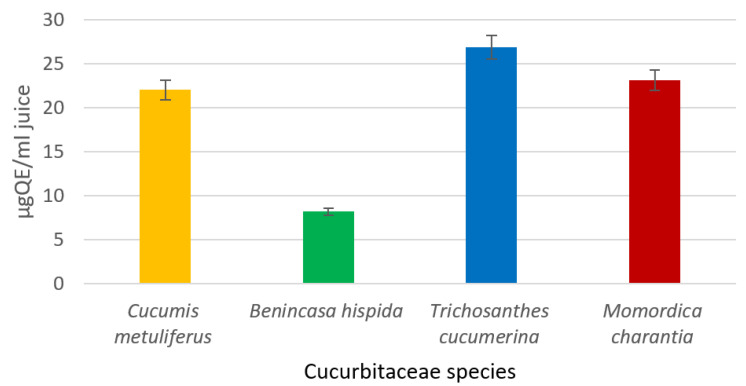
Total flavonoids content of tested samples. The error bars represent ±standard deviation of three replicates.

The found flavonoid contents ranged from 26.82 ± 1.49 µg/mL of quercetin equivalent in the *T. cucumerina* variety to as low as 8.20 ± 0.3 µg/mL in the *B. hispida* variety (Figure 3). Furthermore, the total flavonoid content of the *M. charantia* sample was comparable (23.13 ± 1.33) to that of the *C. metuliferus* sample (22.01 ± 1.29). These results may be due to the content of flavonoid compounds such as catechin hydrate, (−)-epicatechin, phloretin, phloridzin, rutin, quercetin and kaempferol derivatives, and procyanidines.

### 2.4. Ascorbic Acid Quantification

**Figure 4:**
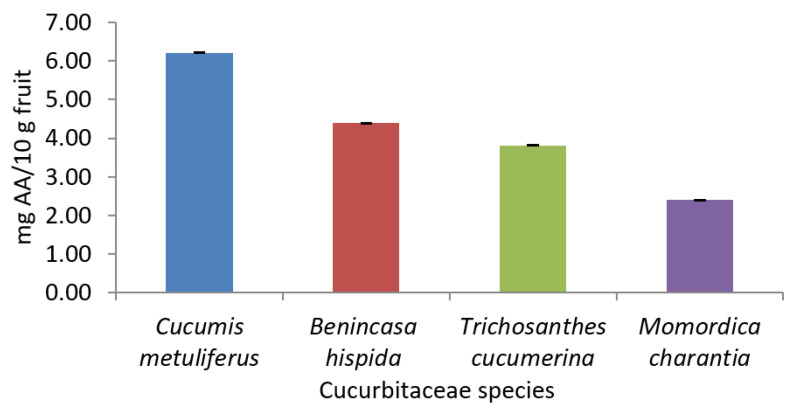
Ascorbic acid (AA) content of samples. The error bars represent ±standard deviation of three replicates.

The ascorbic acid content of the selected species ranged from 2.38 ± 0.01 to 6.32 ± 0.009 mg AA/10 g fruit (Figure 4). *C. metuliferus* showed the highest content of ascorbic acid (6.22 ± 0.01 mg AA/10 g fruit). *M. charantia* displayed a more moderate content of ascorbic acid (2.39 ± 0.02 mg AA/10 g fruit) than those reported in the literature (3.8–85 mg AA/100 g fruit) [38]. Hence, the analyzed fresh fruit from Cucurbitaceae species could be considered as good sources of ascorbic acid.

### 2.5. DPPH Radical Scavenging Activity

The stable radical DPPH absorbs at 517 nm and loses this absorption when it accepts another radical or electron, which leads to its visible discoloration from purple to yellow. The radical is sensitive enough to detect active compounds at low concentrations.

**Figure 5:**
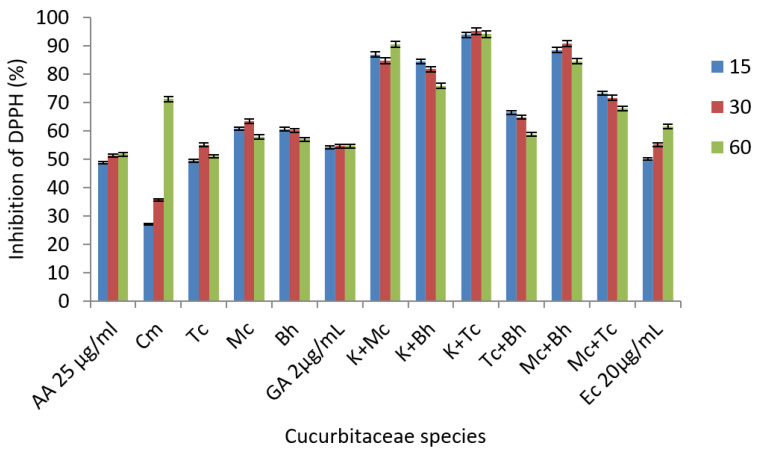
DPPH radical scavenging activity; where AA-ascorbic acid, Cm-*C. metuliferus*, Tc-*T. cucumerina*, Mc-*M. charantia,* Bh-*B. hispida,* GA-gallic acid, Ec-(−)-epicatechin. The error bars represent ±standard deviation of three replicates.

The DPPH radical inhibition assay was used to determine the antioxidant capacity. The individual samples showed a good radical scavenging activity but, more importantly, in this assay, the combination of two species revealed that mixed fruit juices possess a higher antioxidant potential than the individual ones (Figure 5). The DPPH radical inhibition capacity of the tested samples ranged from 20% to 95%, which indicates significant antioxidant activity. The best DPPH activity was obtained for mixed juice of *C. metuliferus* and *T. cucumerina.* The interactions effects of polyphenolic compounds in the juice composition may occur due to the formation of hydrogen bonds between them, leading to a decrease in the ability of hydroxyl groups to inhibit free radicals and then an antagonistic effect may occur. If functional groups such as methoxyl are found in the structure of the compounds, this effect may be reversed and could lead to an increase in antioxidant activity, so a synergistic effect may occur [39]. In the study of Hidalgo et al., pure compounds were studied for their synergistic effect on antioxidant activities. In the DPPH^•^ assay, individual catechin and epicatechin showed lower EC_50_ values when compared with the combination of the same compounds at the same level of concentrations, which means that their combination led to a synergistic antioxidant activity [39]. This may be the explanation for the increased antioxidant effect of the mixtures between *Momordica charantia* or *Thricosanthes cucumerina* which are rich in epicatechin, and *Cucumis metuliferus*, rich in catechin. Muhammad et al. studied the interaction between different combinations of binary mixtures by DPPH^•^ assay, such as epicatechin and gallic acid in various ratios and concluded that they are slightlys synergetic, whereas catechin and gallic acid are slightly to moderately synergetic [40]. The interaction of the juices’ constituents may influence the antioxidant activity of the mixtures, so this may be the reason why some juice mixtures showed a very good to moderate synergetic effect.

### 2.6. ABTS Radical Cation Discoloring Assay

**Figure 6:**
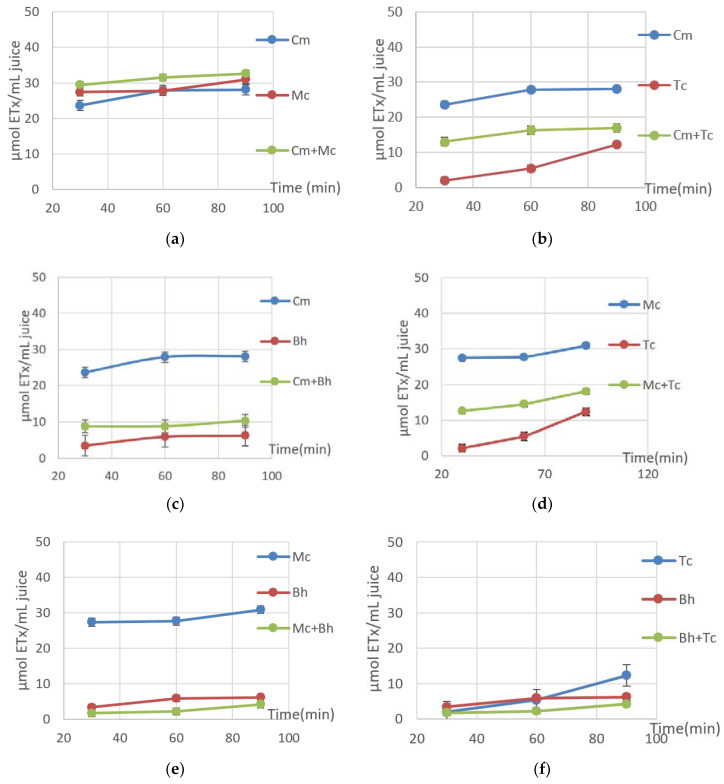
ABTS radical scavenging activity of (**a**) Cm, Mc, Cm + Mc, (**b**) Cm, Tc, Cm + Tc, (**c**) Cm, Bh, Cm + Bh, (**d**) Mc, Tc, Mc + Tc, (**e**) Mc, Bh, Mc + Bh, (**f**) Tc, Bh, Bh + Tc; where Cm-*C. metuliferus*, Tc-*T. cucumerina*, Mc-*M. charantia,* Bh-*B. hispida.*

Our results demonstrate that individual and combined juices of *C. metuliferus* and *M. charantia* have the best synergistic ABTS radical scavenger activity, with values between 29.47 and 32.60 μETx/mL juice (Figure 6). The *B.hispida* and *T. cucumerina* radical scavenger activity was reduced, considering the applied time interval. Two antagonistic mixed juices obtained from *M. charantia* and *B.hispida* and *B.hispida* and *T. cucumerina* (ranging from 2.22 to 6.23 μETx/mL juice) had decreasing radical scavenger activity. On the other hand, the ABTS activity of the mixed juices comprised of *C. metuliferus* and *T. cucumerina, C. metuliferus* and *B. hispida,* or *M. charantia*. and *T. cucumerina* had average values compared to individual juices. Therefore, this analysis is the only method that reflects the antioxidant capacity of fresh juices individually and in combination, with both antagonistic and synergistic effects.

### 2.7. Iron Binding Ability

**Figure 7:**
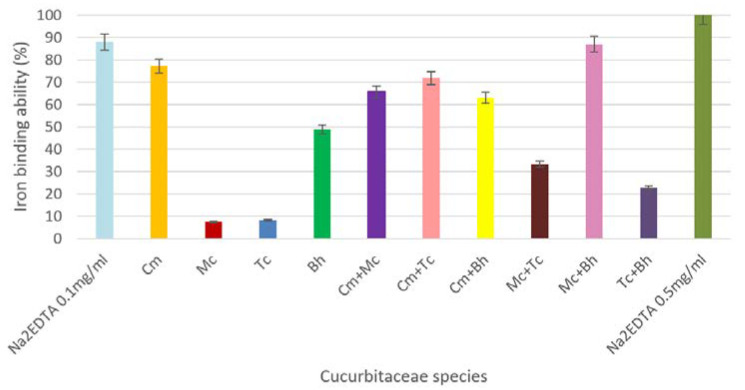
Iron binding ability of the samples; where Cm-*C. metuliferus,* Tc-*T. cucumerina*, Mc-*M. charantia,* Bh-*B. hispida.* The error bars represent ±standard deviation of three replicates.

Iron ions catalyze many processes that lead to the appearance of free radicals and their accumulation in the body can lead to various abnormalities. Therefore, the iron (II) chelating activity of fruit juices could be of great therapeutic significance in neurodegenerative disorders like Alzheimer’s, Parkinson’s diseases, Thalassemia and other anemias [41]. This assay indicates that some bioactive compounds from our juices may have the ability to chelate transition metal ions which could be connected to the type of functional groups from their structure. In our study, the iron chelating abilities of four Cucurbitaceae juices were compared to that of a known strong iron chelator, Na_2_EDTA (Figure 7).

The *C. metuliferus* and *B. hispida* fruit juices showed the highest iron binding ability (77.26 ± 0.39% and 48.93 ± 0.42%, respectively), whereas *M. charantia* showed a low iron binding ability (7.45 ± 0.28%). When combinations of fruit juices assortments were tested, the results revealed that all mixed samples had very good iron binding capacity. The *M. charantia* and *B. hispida* mixed samples exhibited an iron binding ability of 86.95 ± 0.97%, comparable with the one of 0.1 mg/mL Na_2_EDTA positive control (88.02 ± 0.43%).

### 2.8. Total Antioxidant Capacity

**Figure 8:**
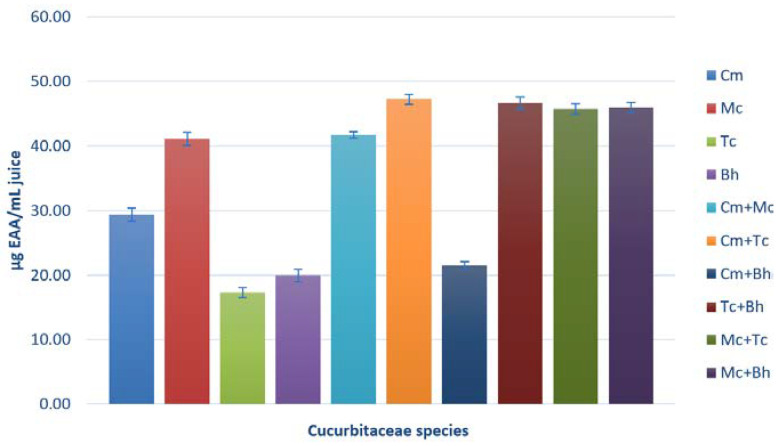
TAC assay; where Cm-*C.metuliferus,* Tc-*T. cucumerina*, Mc-*M. charantia,* Bh-*B. hispida,* AA-ascorbic acid. The error bars represent ±standard deviation of three replicates.

This assay aims to support the idea that the formulation of a combination of selected plants may have a synergistic effect on antioxidant activities [42]. This method, based on the reduction of the molybdenum cation, is a useful quantitative method for evaluating water and fat-soluble compounds with antioxidant capacity (total antioxidant capacity). To our knowledge, this is the first comparative study of TAC in these four species. The analyzed juices exhibited different activities (Figure 8). The *M. charantia* and *C. metuliferus* juices showed the best antioxidant capacity which may be due to the electron-donating existing compounds, such as polyphenols, which can transform free reactive species into non-reactive compounds, which are more stable. The comparison between individual and mixed juices values of TAC assay shows the best antioxidant capacity to reduce molybdenum for *C. metuliferus* and *T. cucumerina* mixed juice.

From the results of all our antioxidant activities, it can be observed that the mixture of two types of juices was more active than the individual ones. It can be concluded that the selected ‘cocktails’ of juices increased the antioxidant effect in a synergistic way which may help human beings to treat various chronic diseases caused by oxidative stresses.

All the results were expressed as the mean values and standard deviation of three replicates for various parameters. No significant differences were found between the groups in all the performed analyses.

**Figure 9:**
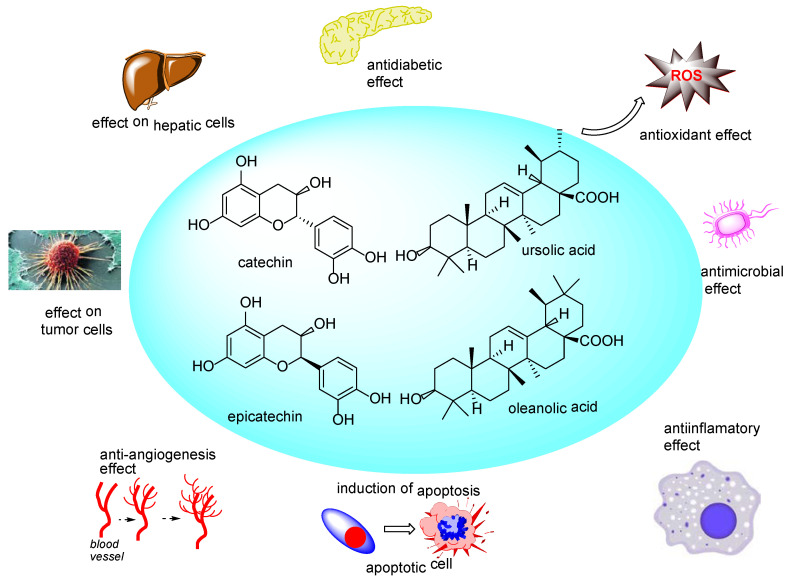
Main components of analyzed Cucurbitaceae juices and their biologic activities.

A summary of the major compounds identified in the analyzed juices of Romanian Cucumber species, together with their known principal biologic activities, demonstrated over the time, is illustrated in Figure 9.

## 3. Materials and Methods

### 3.1. General

The reference compounds (+)-catechin hydrate, (−)-epicatechin, procyanidin A2, procyanidin B2, rutin, quercetin-3-d-galactoside, kaempferol, kaempferol-3-glucoside, quercetin, *p*-coumaric acid, chlorogenic acid, neochlorogenic acid, caffeic acid, gallic acid, *trans*-ferulic acid, ursolic acid and oleanolic acid were purchased from Sigma-Aldrich (Milan, Italy) and are of HPLC grade standards with a purity higher than 99%. All the other reagents and organic solvents were purchased from Fluka (Buchs, Switzerland) and Merck (Darmstadt, Germany).

Fruits of each selected species, i.e., *Momordica charantia* ”Brâncuși” variety*, Cucumis metuliferus* ”Tempus” variety*, Benincasa hispida* ”Zefir” variety, and *Trichosanthes cucumerina* ”Felix” variety, acclimatized in Buzau, Muntenia region, Romania (Southeastern Europe), were obtained at eating ripeness from the Vegetable Research and Development Station (SCDL), Buzau, Romania. The fruits for our research were harvested between 2018 and 2020. The selected species were acclimatized using, as initial material, the seeds of endemic plants from Tibet, Nepal, the Kalahari Desert and China (Hubei University) between 1996 and 2019. The four species involved in the study were grown in a cold greenhouse (without technological heating). The greenhouse was divided into four plots, each species benefiting from a culture space with an area of 500 m^2^. All species benefited from the same crop establishment scheme, with 120 cm between rows and 60 cm between plants per row. For all species, the culture was established in a palisade system, the plants being supported with the help of the strings at the base and directed in the form of a snail to the supporting wires located at a height of 2 m. The care work was general, specific to cucurbits and consisted of water supply when needed, mechanical and manual weeding to control weeds and loosen the soil. After completing the acclimatization stage, the species were subjected to intensive breeding works, obtaining genotypes with distinct phenotypic expressiveness, of which the four varieties were registered in the Official Catalog of Cultivated Plants in Romania.

The fruit juices were prepared as described by Mahmoud et al. (2017) with slight modifications [43]. Briefly, fruits without blemishes or damage (1 kg) were selected, then they were washed well and squeezed using a commercial juice machine (Tefal Frutelia ZE420D38, 11, 400 W). The fresh juice was centrifuged at 6000× rpm for 20 min. The clear supernatant was taken as 100% test sample and stored at 4 °C for further analysis, for a maximum of 48 h. The juice combinations were obtained by mixing the juices in a volumetric ratio of 1:1.

### 3.2. HPLC Analysis of Juices Constituents

High-performance liquid chromatography with photodiode-array (HPLC-DAD) studies were performed using a Hewlett-Packard HP-1090 Series II (Palo Alto, CA, USA), equipped with a vacuum degasser, a binary pump, an autosampler and a model 1046A HP photodiode array detector (DAD) and a mass spectrometer detector Trap SL (Bruker, Billerica, MA, USA) equipped with an electrospray ionization (ESI) source. The chromatographic separation was accomplished on a Synergi Polar-RP C18 (4.6 mm × 250 mm, 4 µm) analytical column from Phenomenex (Chesire, UK). The column was preceded by a security cartridge. The mobile phase for HPLC-DAD analyses was a mixture of water with 0.1% formic acid (*v*/*v*) and methanol with 0.1% formic acid (*v*/*v*), flowing at 1 mL/min in gradient conditions: 0–15 min, 20% B; 15–45 min, 100% B; 45–60 min, 20% B. The column temperature was set at 30 °C and the injection volume was 10 µL [44]. UV-visible spectra were recorded in the range 210–520 nm for the 17 compounds, where 272 nm was used for gallic acid, 325 nm for neochlorogenic acid, chlorogenic acid, caffeic acid, *p*-coumaric acid and *trans*-ferulic acid, 280 nm for (+)-catechin hydrate, (−)-epicatechin, and; 230 nm for procyanidin A2 and procyanidin B2, 265 nm for rutin, quercetin-3-d-galactoside and kaempferol-3-glucoside, 365 nm for kaempferol and quercetin, 210 nm for ursolic and oleanolic acids (Figures S1–S28 from the Supplementary Materials). The quantification of the different analytes was carried out using the validated method of the seventeen compounds, as described in Wandjou et al. [44]. Injection of 1–50 mg/L standard solutions at five different concentrations (i.e., 1, 5, 10, 25, and 50 mg/L) into the HPLC/DAD system was done to construct the calibration curves of the analyzed compounds. The correlation coefficient (R^2^) of all the calibration curves was greater than 0.99, displaying the good linearity of the method. The quantification of the different analytes was carried out using the single calibration curve of the seventeen analytes [44].

The fresh juices taken as the test sample were centrifuged at 9000× rpm for 20 min, and then the clear supernatant was filtrated before the analysis. The analyzes were performed in triplicate.

### 3.3. Total Polyphenol Content Assay (TPC)

The total polyphenol content determination was completed using an adapted microspectrophotometric laboratory method [45] based on Folin-Ciocalteu assay. The absorbance was recorded at 760 nm by a 96-well plate analysis with a multiplate reader (Tecan Pro 200, Tecan Trading AG, Männedorf, Switzerland). Freshly prepared gallic acid solutions of known concentrations (0.97–500 µg/mL) were used to prepare the standard curve. Briefly, 10 µL of fresh juice from each test sample and 25 µL of Folin-Ciocalteu reagent were mixed and stored at room temperature for 5 min. The next step was the addition of 25 µL of a 20% Na_2_CO_3_ solution and 140 µL of ultrapure water and stored for another 30 min at room temperature. Finally, the absorbance was recorded at 760 nm and the results are expressed as mg gallic acid equivalent/100 mL fresh juice (mg GAE/mL fresh juice). For each sample, a blank was prepared by replacing the Folin-Ciocalteu reagent with ultrapure water [46]. All the experiments were done in triplicate.

### 3.4. Total Flavonoids Content Assay

Aluminium chloride was used to quantify the total flavonoid content by a 96-well plate analysis, as per the method previously published [12,45,46]. Quercetin was used as a reference standard (40–0.078 µg/mL). An amount of 100 µL test sample was stored at room temperature for 15 min with 100 µL of a 2% aqueous solution of aluminum chloride and the absorbance was recorded at 415 nm. All the experiments were done in triplicate.

### 3.5. Ascorbic Acid Quantification

Ascorbic acid presents a significant importance for human health, is a strong reducing agent and can be found in vegetables and fruits. For the estimation of the ascorbic acid content, various spectrophotometric methods have been reported. This methods are based on oxidizing agents such as ferric salts, methylene blue, potassium permanganate or phenol indophenol [47,48,49]. A microplate assay based on potassium permanganate, as oxidizing agent, was applied in an acid medium to quantify ascorbic acid and therefore to explore the health-promoting properties of the juices obtained from the tested fruits. Freshly prepared samples were obtained by weighing 10 g of fruit sample in a 50 mL beaker, then adding meta-Phosphoric acid (3%) to avoid the oxidation of the ascorbic acid and mixing well to obtain the fruit juice. All the samples were filtered and the filtrate was transferred to 200 volumetric flasks and then completed to the mark with meta-Phosphoric acid (3%). Blank sample was prepared by replacing the fruit samples with distilled water. Standard solution of ascorbic acid was prepared in meta-Phosphoric acid (3%) to obtain a 1-mg/mL stock solution. A series of dilutions 0.2, 0.1, 0.05, 0.025, 0.0125 was prepared from the stock solution. A 0.1-mg/mL KMnO_4_ solution was prepared in H_2_SO_4_ solution (5.0 M). An amount of 20 µL of sample was mixed with 180 µL of 0.1 mg/mL acid solution of KMnO_4_ and left to sit in the dark for 5 min. The absorbance of the samples was read at 530 nm. In this assay, the ascorbic acid consumes the potassium permanganate (violet solution), resulting in a decrease in the absorbance of 530 nm. All the experiments were done in triplicate.

### 3.6. 2,2-Diphenyl-1-Picrylhydrazyl Radical Scavenging Activity Assay

Antioxidant activity has been reported in various manners, such as the percentage of utilized reagent and the percentage of oxidation inhibition. Antioxidant compounds can be water-soluble, insoluble, or bound to the cell walls. As such, the efficiency of extraction is an important factor in quantifying a plant’s antioxidant activity [50]. The antioxidative effects of the tested samples were estimated by DPPH (1,1-Diphenyl-2-Picrylhydrazyl) radical scavenging assay. The DPPH-free radical has a maximum absorption at 517 nm, which gives it a purple color. The color shifts from purple to yellow, resulting in a reduced form of DPPH. In a 96-well plate, 100 µL of test sample was mixed and homogenized with 100 µL of a 100 µg/mL DPPH methanolic solution. In this assay, two by two, mixed juices samples were subjected to this determination in equal volumes. Fresh methanolic DPPH solution served as the control [51]. The resulting solutions were then stored in the dark at room temperature. The absorbance values at 517 nm were recorded after 15, 30 and 60 min. An amount of 100 µL methanol mixed with 100 µL of test samples were used as blank. The radical scavenging activities of three standards were assed using this protocol. Gallic acid, (−)-epicatechin and ascorbic acid were dissolved and diluted into different concentrations in methanol, and then they were used as references. All the experiments were done in triplicate.

The DPPH inhibition percentage, which relates to the antioxidant activity of quercetin and the juices, was calculated using the following formula:

% inhibition of DPPH radical = (1 − A_s_/A_c_) × 100 (1)

where A_c_ is the absorbance of the control and A_s_ is the absorbance of the sample.

### 3.7. ABTS Radical Cation Discolouring Assay

2,2-Azino-bis(3-ethylbenzothiazoline-6-sulfonic acid (ABTS) radical cation scavenging was determined as previously described in the literature [45,46], with slight modifications. The ABTS^+^ solution was prepared and then stored for 16 h in dark conditions at room temperature. The solution was diluted with absolute ethanol until the absorbance at 734 nm was 0.700 ± 0.02, and then it was used as the ABTS^+^ reagent. The absorbance of the final reacting solution was measured at 734 nm in a 96-well plate. An amount of 100 µL fresh juice of the four Cucurbitaceae species with 100 µl of ABTS^+^ reagent was mixed and stored for 30, 60 and 90 min. The final results were expressed by means of using a Trolox calibration curve (calibration curve from different concentrations: 50–0.25 µM). All the experiments were done in triplicate.

### 3.8. Determination of Iron Binding Ability

The ferrous ion was monitored by measuring the formation of a red ferrous ion-ferrozine complex at 562 nm as previously described in the literature [52], adapted to the 96-well plate analysis. If the formed ferrous ion-ferrozine complex is stronger, the absorbance at 562 nm is higher, and if the ferrous iron binding strength of the chelator is weaker, the absorbance is smaller. An amount of 225 µL test sample was mixed with 10 µL ferrous sulphate (2 mM), then were stored at room temperature for 5 min at room temperature and 15 µL ferrozine (0.2 mM) was added to start the reaction. A blank was prepared by using distilled water instead of ferrozine. The resulting mixture was mixed and left to stand for 10 min at room temperature and then the absorbance was measured at 562 nm. EDTA was used as standard. Distilled water was used as control. All the experiments were done in triplicate.

The ferrous ion binding ability of the iron chelators was calculated using the formula below:

Iron binding ability (%) = (A_c_ − [A_s_ − A_b_])/A_c_ × 100% (2)

where A_c_ is the absorbance of the control, A_b_ is the blank for each sample and A_s_ is the absorbance of the iron chelator.

### 3.9. Total Antioxidant Capacity

This assay is based on the reduction of Mo (VI) to Mo (V) and subsequent formation of a green Mo (V)-complex at acidic pH levels [53]. For total antioxidant capacity assay, 0.1 mL test sample was mixed in Eppendorf tubes with 1 mL of reagent solution (0.6 M sulfuric acid, 28 mM sodium phosphate, and 4 mM ammonium molybdate). Then, they were incubated at 95 °C for 90 min. After the mixture had cooled to room temperature, the absorbance of each solution was measured at 695 nm. Total antioxidant capacity is reported as equivalents of ascorbic acid. All the experiments were done in triplicate.

### 3.10. Statistical Analysis

The results are expressed as mean values and standard deviation of three replicates for various parameters unless otherwise mentioned. Significant differences among results were identified by means of analysis of variance (ANOVA). Results with *p* values ≤ 0.05 were deemed statistically significant.

## 4. Conclusions

In this paper, we present the first comparative study of four juices from Romanian Cucurbitaceae varieties, performing HPLC quantification of major components together with evaluation of their different antioxidant and iron binding activities. The synergistic/antagonistic effect of the juice mixture was also studied. The most abundant compounds shown by HPLC analysis were the two triterpenes, oleanolic and ursolic acids, as well as catechin, epicatechin with the exception of *Benincasa hispida* which is rich in gallic acid. One of the highest total antioxidant capacities was obtained for *M. charantia* juice but a synergistic effect was obtained for *C. metuliferus* and *T*. *cucumerina* mixed juice. It is already known that the presence of phenolic compounds and flavonoids offers plants antioxidant potential and our results highlight this property. These results suggest that a synergistic antioxidant effect can be achieved by accumulating the combined effects of all antioxidant compounds. Our results will help avoid unwanted side effects due to higher amounts of juice consumed from a single variety of fruit, and therefore, play an important role for helping to treat various diseases. The studies on the properties of combined juices also suggest that for further analysis, it will be crucial to select the species that may be of further importance in the study of biological activity synergism.

## Figures and Tables

**Table 1:** Chemical composition of fresh juices (expressed as mg kg^−1^ ± standard deviation).

Compounds	RT *	*M. charantia*	*C. metuliferus*	*B. hispida*	*T. cucumerina*
Hydroxybenzoic acids					
Gallic acid	5.9	3386.78 ± 1.60 ^a^	5.76 ± 0.31 ^d^	525.24 ± 0.80 ^b^	21.59 ± 0.27 ^c^
Catechin	17.6	201.78 ± 1.45 ^b^	928.74 ± 0.97 ^a^	17.47 ± 0.48 ^c^	n.d.
Epicatechin	23.9	555.22 ± 4.66 ^a^	17.66 ± 0.42 ^c^	24.24 ± 0.42 ^b^	n.d.
Procianidin B2	24.3	38.48 ± 0.66	n.d.	n.d.	255.46 ± 3.60
Procianidin A2	29.9	16.37 ± 0.45	n.d.	n.d.	n.d.
Flavonols					
Rutin	31.47	29.79 ± 0.71 ^b^	33.78 ± 0.55 ^a^	12.87 ± 0.16 ^d^	19.58 ± 0.39 ^c^
Quercetin	35.8	80.86 ± 0.59 ^a^	5.96 ± 0.06 ^b^	4.00 ± 0.00 ^b^	4.64 ± 0.11 ^b^
Quercetin-3-d-galactoside	32.00	15.45 ± 0.23 ^a^	2.35 ± 0.05 ^c^	3.49 ± 0.10 ^b^	1.89 ± 0.08 ^c^
Kampferol-3-glucoside	33.6	80.86 ± 0.59 ^a^	19.61 ± 0.41 ^b^	n.d.	19.90 ± 0.12 ^b^
Kampferol	37.8	2.78 ± 0.11 ^b^	4.32 ± 0.31 ^a^	n.d.	3.59 ± 0.27 ^a,b^
Hydrocinnamic acids					
Neochlorogenic acid	10.58	9.11 ± 9.30	4.68 ± 0.09	n.d.	n.d.
Chlorogenic acid	22.3	n.d.	n.d.	n.d.	n.d.
Caffeic acid	22.9	n.d.	5.56 ± 0.26	n.d.	4.97 ± 1.88
*p*-Coumaric acid	28.9	4.07 ± 0.37 ^b^	6.99 ± 0.57 ^b^	n.d.	13.44 ± 1.56 ^a^
trans-Ferulic acid	30.5	6.20 ± 2.14 ^a^	n.d.	6.66 ± 0.10 ^a^	2.98 ± 0.76 ^b^
Triterpenes					
Oleanolic acid	45.8	238.10 ± 1.45 ^c^	347.67 ± 1.45 ^a^	326.16 ± 2.17 ^b^	352.79 ± 7.97 ^a^
Ursolic acid	45.9	195.44 ± 1.37 ^c^	193.92 ± 2.34 ^c^	377.35 ± 5.08 ^a^	243.78 ± 4.88 ^b^
Total		4861.3 ±2.26	1577.01 ± 0.65	1297.48 ± 1.66	944.62 ± 2.47

* RT means retention time, n.d. means not detected. Mean values on the same line marked with different letters (a,b,c,d) are significantly different according to the Duncan test (*p* < 0.05).

## Supplementary Materials

The following are available online, Figure S1–S7: HPLC chromatogram of *M. charantia* sample at 210, 230, 265, 272, 280, 325 and 365 nm, Figure S8–S14: HPLC chromatogram of *T. cucumerina* sample at 210, 230, 265, 272, 280, 325 and 365 nm, Figure S15–S21: HPLC chromatogram of B. hispida sample at 210, 230, 265, 272, 280, 325 and 365 nm, Figure S22–S28: HPLC chromatogram of *C. metuliferus* sample at 210, 230, 265, 272, 280, 325 and 365 nm.

## References

[1] Harborne J.B. (1998). Phytochemical Methods a Guide to Modern Tecniques of Plant Analysis.

[2] Mao S., Wang K., Lei Y., Yao S., Lu B., Huang W. (2017). Antioxidant synergistic effects of Osmanthus fragrans flowers with green tea and their major contributed antioxidant compounds. Sci. Rep..

[3] Bisognin D.A. (2002). Origin and evolution of cultivated cucurbits. Ciência Rural.

[4] Ajuru M., Nmom F. (2017). A review on the economic uses of species of Cucurbitaceae and their sustainability in Nigeria. Am. J. Plant Biol..

[5] Rolnik A., Olas B. (2020). Vegetables from the Cucurbitaceae family and their products: Positive effect on human health. Nutrition.

[6] Rajasree R.S., Sibi P.I., Francis F., William H. (2016). Phytochemicals of cucurbitaceae family—A review. Int. J. Pharmacogn. Phytochem. Res..

[7] Grover J.K., Yadav S.P. (2004). Pharmacological actions and potential uses of Momordica charantia: A review. J. Ethnopharmacol..

[8] Aeri V., Kaushik U., Mir S.R. (2015). Cucurbitacins—An insight into medicinal leads from nature. Pharmacogn. Rev..

[9] Gao H., Wen J.-J., Hu J.-L., Nie Q.-X., Chen H.-H., Xiong T., Nie S.-P., Xie M.-Y. (2019). Fermented Momordica charantia L. juice modulates hyperglycemia, lipid profile, and gut microbiota in type 2 diabetic rats. Food Res. Int..

[10] Wang H.-Y., Kan W.-C., Cheng T.-J., Yu S.-H., Chang L.-H., Chuu J.-J. (2014). Differential anti-diabetic effects and mechanism of action of charantin-rich extract of Taiwanese Momordica charantia between type 1 and type 2 diabetic mice. Food Chem. Toxicol..

[11] Krishnaiah D., Sarbatly R., Nithyanandam R. (2011). A review of the antioxidant potential of medicinal plant species. Food Bioprod. Process..

[12] Tan S.P., Stathopoulos C.E., Parks S., Roach P. (2014). An Optimised Aqueous Extract of Phenolic Compounds from Bitter Melon with High Antioxidant Capacity. Antioxidants.

[13] Kenny O., Smyth T.J., Hewage C.M., Brunton N. (2013). Antioxidant properties and quantitative UPLC-MS analysis of phenolic compounds from extracts of fenugreek (Trigonella foenum-graecum) seeds and bitter melon (Momordica charantia) fruit. Food Chem..

[14] Yeşilada E., Gürbüz I., Shibata H. (1999). Screening of Turkish anti-ulcerogenic folk remedies for anti-Helicobacter pylori activity. J. Ethnopharmacol..

[15] Benzioni A., Mendlinger S., Ventura M., Huyskens S., Janick J., Simon J.E. (1993). Germination, Fruit Development, Yield, and Postharvest Characteristics of Cucumis metuliferus. New Crops.

[16] Jimam N., Wannang N., Omale S., Gotom B. (2010). Evaluation of the Hypoglycemic Activity of Cucumis metuliferus (Cucurbitaceae) Fruit Pulp Extract in Normoglycemic and Alloxan-Induced Hyperglycemic Rats. J. Young Pharm..

[17] Matsusaka Y., Kawabata J. (2010). Evaluation of Antioxidant Capacity of Non-Edible Parts of Some Selected Tropical Fruits. Food Sci. Technol. Res..

[18] Breithaupt D.E., Wirt U., Bamedi A. (2002). Differentiation between Lutein Monoester Regioisomers and Detection of Lutein Diesters from Marigold Flowers (Tagetes erectaL.) and Several Fruits by Liquid Chromatography−Mass Spectrometry. J. Agric. Food Chem..

[19] Tennekoon K.H., Jeevathayaparan S., Angunawala P., Karunanayake E.H., Jayasinghe K. (1994). Effect of Momordica charantia on key hepatic enzymes. J. Ethnopharmacol..

[20] Arr K.L., Xia Y.E.I., Bhattarai N.K. (2007). Nutritional Data of Benincasa hlspida (Cucurbitaceae) 1.

[21] Lee K.-H., Choi H.-R., Kim C.-H. (2005). Anti-angiogenic effect of the seed extract of Benincasa hispida Cogniaux. J. Ethnopharmacol..

[22] Bhalodia Y., Kanzariya N., Patel R., Patel N., Vaghasiya J., Jivani N., Raval H. (2009). Renoprotective activity of benincasa cerifera fruit extract on ischemia/reperfusion-induced renal damage in rat. Iran. J. Kidney Dis..

[23] Sun X., Baldwin E., Plotto A., Cameron R.G., Manthey J., Dorado C., Bai J. (2018). The effect of cultivar and processing method on the stability, flavor, and nutritional properties of winter melon juice. LWT.

[24] Huang H.-Y., Huang J.-J., Tso T.K., Tsai Y.-C., Chang C.-K. (2004). Antioxidant and angiotension-converting enzyme inhibition capacities of various parts ofBenincasa hispida (wax gourd). Food/Nahrung.

[25] Kumar A., Vimalavathini R. (2004). Possible anorectic effect of methanol extract of Benincasa hispida (Thunb). Cogn. Fruit. Indian J. Pharmacol..

[26] Doshi G., Nalawade V., Mukadam A.S., Chaskar P.K., Zine S.P., Somani R.R., Une H.D. (2015). Structural elucidation of chemical constituents from Benincasa hispida seeds and Carissa congesta roots by gas chromatography: Mass spectroscopy. Pharmacogn. Res..

[27] Devi N. (2017). Medicinal Values of *Trichosanthus cucumerina* L. (Snake Gourd)—A Review. J. Pharm. Res. Int..

[28] Igwe C., Ojiako O. (2008). The Nutritive, Anti-Nutritive and Hepatotoxic Properties of Trichosanthes anguina (Snake Tomato) Fruits from Nigeria. Pak. J. Nutr..

[29] Arawwawala M., Thabrew I., Arambewela L. (2009). Antidiabetic activity of Trichosanthes cucumerina in normal and streptozotocin–induced diabetic rats. Int. J. Biol. Chem. Sci..

[30] Arawwawala L., Thabrew M., Arambewela L. (2010). Gastroprotective activity of Trichosanthes cucumerina in rats. J. Ethnopharmacol..

[31] Mendiratta M., Gupta S. (2017). Exploring the Role of Phytochemicals and Antioxidants on Antihyperglycemic Potentials of Indian Medicinal Plants. Int. J. Gen. Med. Pharm..

[32] Anila L., Vijayalakshmi N.R. (2000). Beneficial effects of flavonoids from Sesamum indicum, Emblica officinalis and Momordica charantia. Phytother. Res..

[33] Saeed F., Afzaal M., Niaz B., Arshad M.U., Tufail T., Hussain M.B., Javed A. (2018). Bitter melon (Momordica charantia): A natural healthy vegetable. Int. J. Food Prop..

[34] Ferrara L. (2018). A fruit to discover: Cucumis metuliferus E.Mey Ex Naudin (Kiwano). Clin. Nutr. Metab..

[35] Fatariah Z., Zulkhairuazha T.Y.T., Rosli W.I.W. (2014). Quantitative HPLC analysis of gallic acid in benincasa hispida prepared with different extraction techniques. Sains Malays..

[36] Al-Ishaq R.K., Abotaleb M., Kubatka P., Kajo K., Büsselberg D. (2019). Flavonoids and Their Anti-Diabetic Effects: Cellular Mechanisms and Effects to Improve Blood Sugar Levels. Biomolecules.

[37] Novotny L., Vachálková A., Biggs D. (2001). Ursolic acid: An anti-tumorigenic and chemopreventive activity. Minireview. Neoplasma.

[38] Choo W.S., Yap J.Y., Chan S.Y. (2014). Antioxidant properties of two varieties of bitter gourd (Momordica charantia) and the effect of blanching and boiling on them. Pertanika J. Trop. Agric. Sci..

[39] Hidalgo M., Sánchez-Moreno C., De Pascual-Teresa S. (2010). Flavonoid–flavonoid interaction and its effect on their antioxidant activity. Food Chem..

[40] Muhammad D.R.A., Praseptiangga D., Van De Walle D., Dewettinck K. (2017). Interaction between natural antioxidants derived from cinnamon and cocoa in binary and complex mixtures. Food Chem..

[41] Sudan R., Bhagat M., Gupta S., Singh J., Koul A. (2014). Iron (FeII) Chelation, Ferric Reducing Antioxidant Power, and Immune Modulating Potential ofArisaema jacquemontii(Himalayan Cobra Lily). BioMed Res. Int..

[42] Jain D.P., Pancholi S.S., Patel R. (2011). Synergistic antioxidant activity of green tea with some herbs. J. Adv. Pharm. Technol. Res..

[43] Mahmoud M.F., Ashry F.E.Z.Z.E., El Maraghy N.N., Fahmy A. (2017). Studies on the antidiabetic activities of Momordica charantia fruit juice in streptozotocin-induced diabetic rats. Pharm. Biol..

[44] Wandjou J.G.N., Mevi S., Sagratini G., Vittori S., Dall’Acqua S., Caprioli G., Lupidi G., Mombelli G., Arpini S., Allegrini P. (2019). Antioxidant and Enzyme Inhibitory Properties of the Polyphenolic-Rich Extract from an Ancient Apple Variety of Central Italy (Mela Rosa dei Monti Sibillini). Plants.

[45] Balli D., Cecchi L., Khatib M., Bellumori M., Cairone F., Carradori S., Zengin G., Cesa S., Innocenti M., Mulinacci N. (2020). Characterization of Arils Juice and Peel Decoction of Fifteen Varieties of Punica granatum L.: A Focus on Anthocyanins, Ellagitannins and Polysaccharides. Antioxidants.

[46] Cudălbeanu M., Ghinea I.O., Furdui B., Dah-Nouvlessounon D., Raclea R.-C., Costache T., Cucolea I.E., Urlan F., Dinica R., Cudalbeanu M. (2018). Exploring New Antioxidant and Mineral Compounds from Nymphaea alba Wild-Grown in Danube Delta Biosphere. Molecules.

[47] Ojukwu U.P., Nwobi S.C. (2017). Determination of ascorbic acid content of some local fruits in Nigeria. Anal. Chem. Indian J..

[48] Zanini D.J., Silva M.H., Aguiar-Oliveira E., Mazalli M.R., Kamimura E.S., Maldonado R.R. (2018). Spectrophotometric Analysis of Vitamin C in Different Matrices. Eur. Int. J. Sci. Technol..

[49] Grudpan K. (1999). Flow injection spectrophotometric or conductometric determination of ascorbic acid in a vitamin C tablet using permanganate or ammonia. Talanta.

[50] Saleem A., Saleem M., Akhtar M.F. (2020). Antioxidant, anti-inflammatory and antiarthritic potential of Moringa oleifera Lam: An ethnomedicinal plant of Moringaceae family. S. Afr. J. Bot..

[51] Alam N., Bristi N.J. (2013). Rafiquzzaman Review on in vivo and in vitro methods evaluation of antioxidant activity. Saudi Pharm. J..

[52] Adjimani J.P., Asare P. (2015). Antioxidant and free radical scavenging activity of iron chelators. Toxicol. Rep..

[53] Govindarajan R., Rastogi S., Vijayakumar M., Shirwaikar A., Rawat A.K.S., Mehrotra S., Pushpangadan P. (2003). Studies on the antioxidant activities of Desmodium gangeticum. Biol. Pharm. Bull..

