# Supplementary material for: Comparative Study of the Chemical Compositions and Antioxidant Activities of Fresh Juices from Romanian Cucurbitaceae Varieties
Source: Molecules. 2020 Nov 23;25(22):5468. doi: 10.3390/molecules25225468 (PMC7700580; doi:10.3390/molecules25225468)

molecules

MDPI

Supplementary Materials:

Comparative study of the chemical compositions and antioxidant activities of fresh juices from Cucurbitaceae species grown in Romania

Anna Cazanevscaia (Busuioc) [1], Andreea-Veronica Dediu (Botezatu) [1], Bianca Furdui [1,*], Costel Vanatoru [3], Filippo Maggi [2], Giovanni Caprioli [2] and Rodica-Mihaela Dinica [1,*]

[1] Department of Chemistry, Physics and Environment, "Dunărea de Jos" University of Galati, 111 Domnească Street, 800201 Galati, Romania; email: anna.cazanevscaia@ugal.ro (A.C.B.); veronica.dediu@ugal.ro (A.V.B.); bianca.furdui@ugal.ro (B.F.);

[2] School of Pharmacy, University of Camerino, Via S. Agostino 1, 62032 Camerino, Italy; filippo.maggi@unicam.it ; giovanni.caprioli@unicam.it

[3] Buzău Vegetable Research and Development Station, 23, Mesteacănului Street, 120 024, Buzau, Romania; costel_vinatorul@yahoo.com

* Correspondence: rodica.dinica@ugal.ro (R.-M.D.); bianca.furdui@ugal.ro (B.F.)

M. charantia

Figure S1. HPLC chromatogram of M. charantia sample. 210nm Ursolic acid (RT-45.9) and Oleanolic acids (RT-45.8)

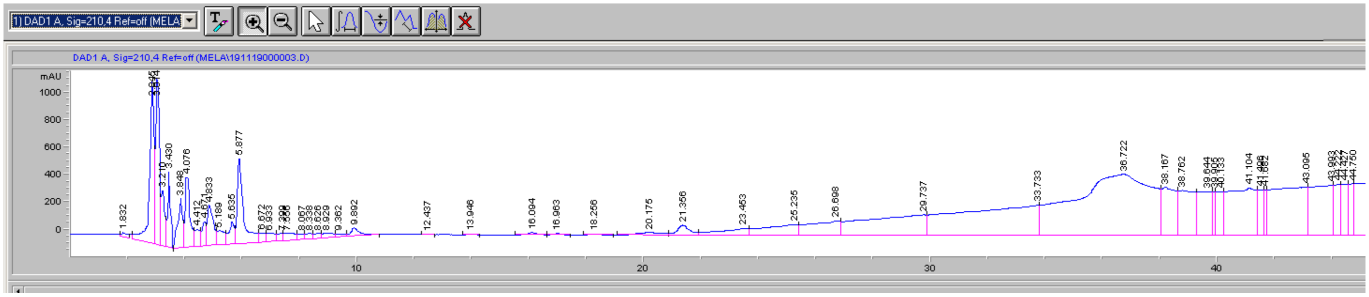

Figure S2. HPLC chromatogram of M. charantia sample. 230nm Procyanidin A2 (RT-24.3) and Procyanidin B2 (RT-29.9)

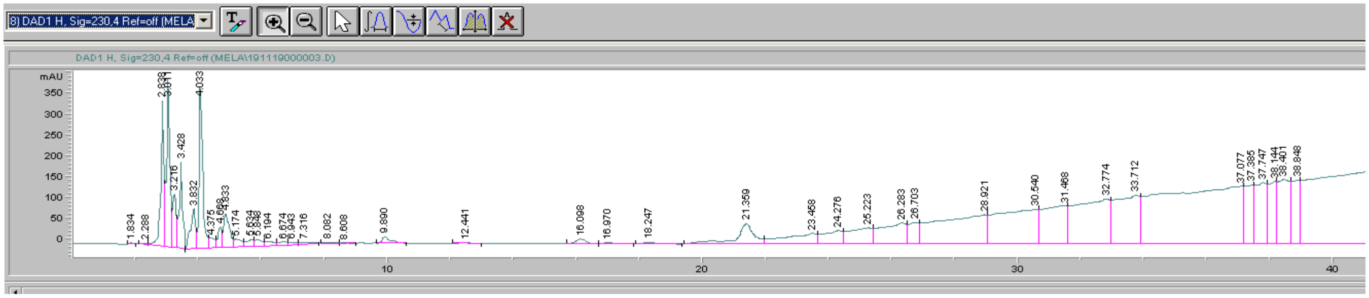

molecules

MDPI

Figure S3. HPLC chromatogram of M. charantia sample. 265nm Rutin (RT-31.47), Quercetin-3-D-galactoside (RT-32) and Kaempferol-3-glucoside (RT-33.6)

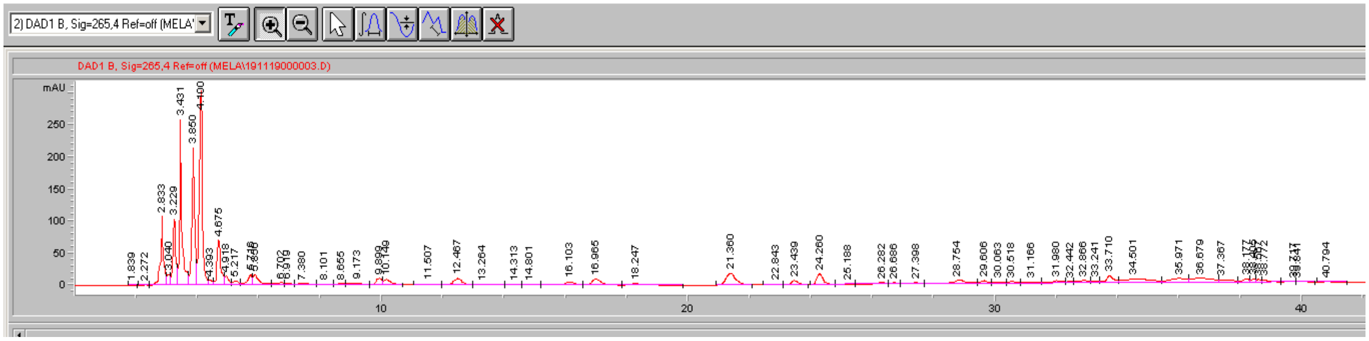

Figure S4. HPLC chromatogram of M. charantia sample.272nm - Gallic acid RT -5.9

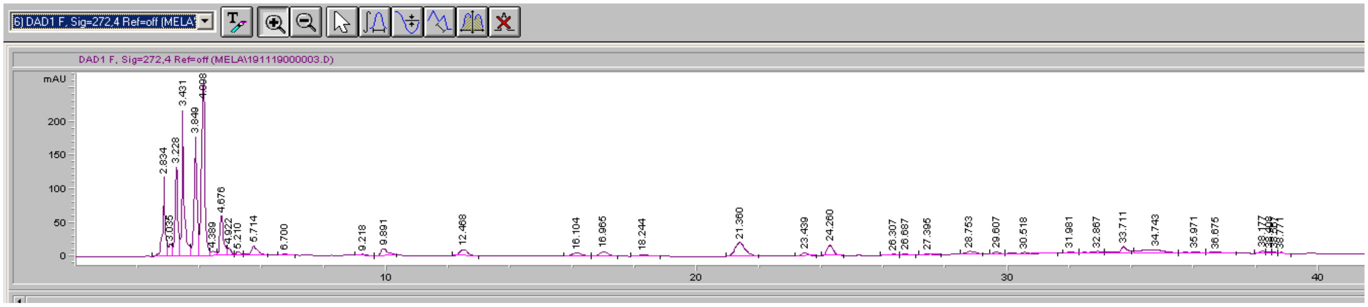

Figure S5. HPLC chromatogram of M. charantia sample. 280nm (+)-catechin hydrate (RT-17.6), (-)-epicatechin (RT-23.9)

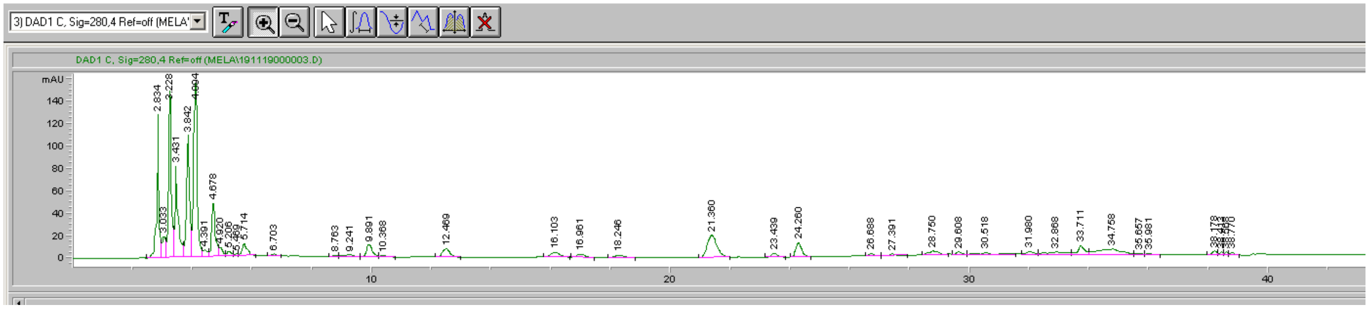

Figure S6. HPLC chromatogram of M. charantia sample. 325nm Neochlorogenic acid (RT-10.58), Chlorogenic acid (RT-22.3), Caffeic acid (RT-22.9), p-Coumaric acid (RT- 28.9) and trans-ferulic acid (RT-30.5)

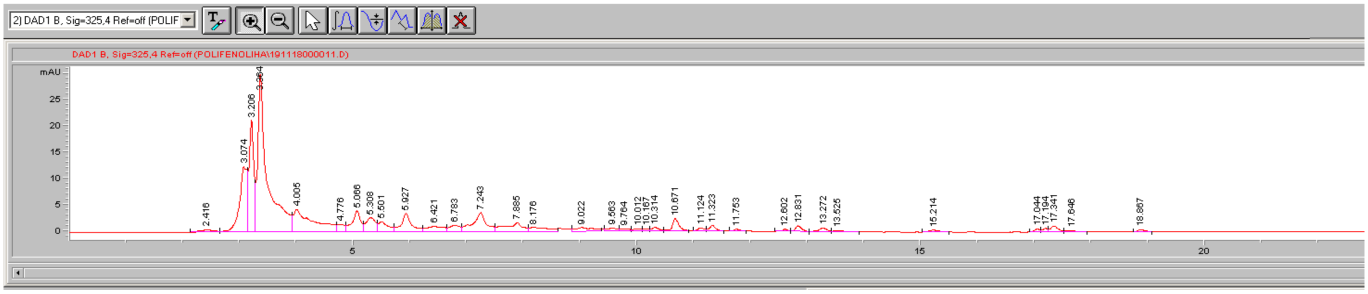

molecules

MDPI

Figure S7. HPLC chromatogram of M. charantia sample. 365nm Kaempferol (RT-37.8) and Quercetin (RT-35.8)

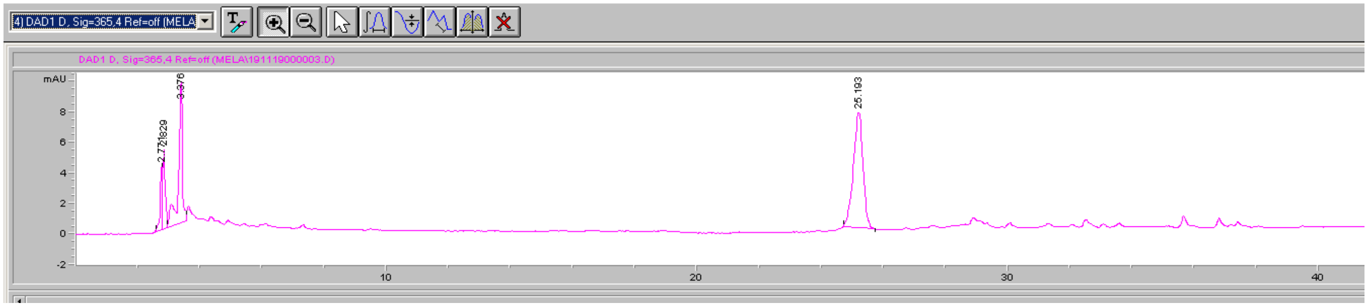

Figure S8. HPLC chromatogram of T. cucumerina sample. 210nm Ursolic acid (RT-45.9) and Oleanolic acids (RT-45.8)

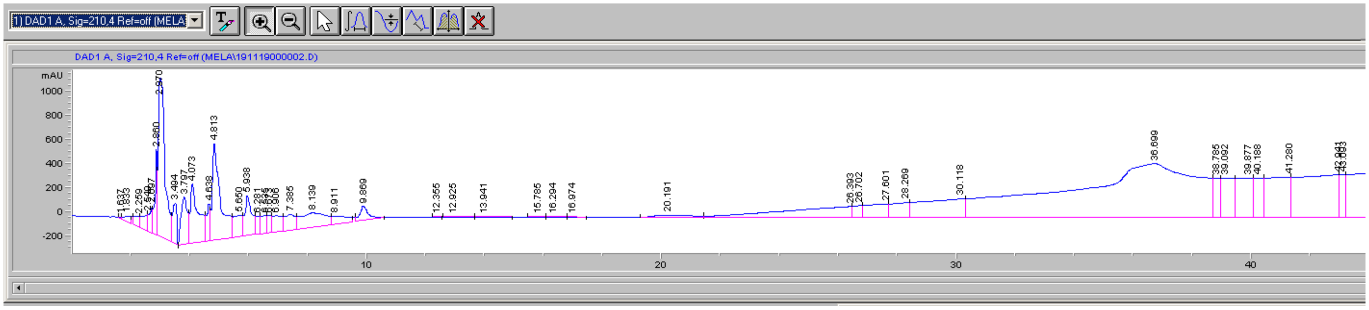

Figure S9. HPLC chromatogram of T. cucumerina sample. 230nm Procyanidin A2 (RT-24.3) and Procyanidin B2 (RT-29.9)

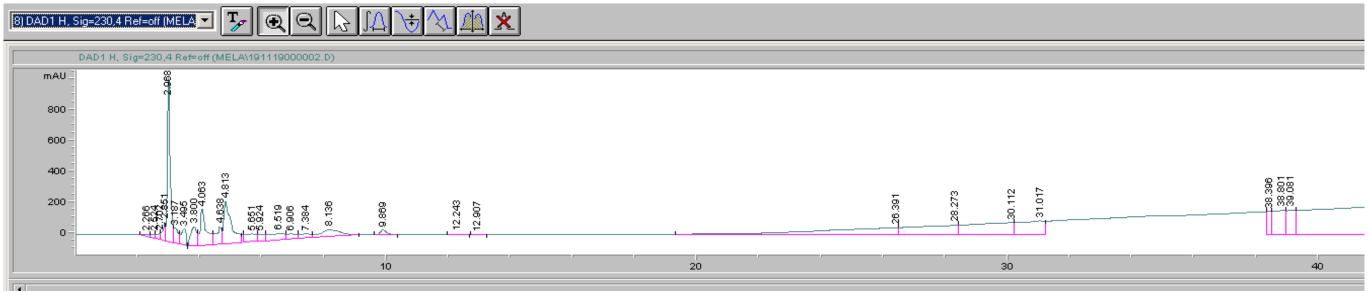

Figure S10. HPLC chromatogram of T. cucumerina sample. 265nm Rutin (RT-31.47), Quercetin-3-D-galactoside (RT-32) and Kaempferol-3-glucoside (RT-33.6)

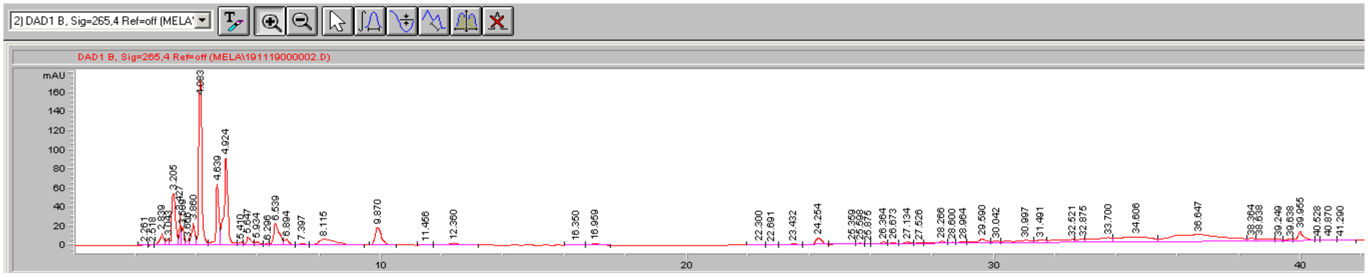

molecules

MDPI

Figure S11. HPLC chromatogram of T. cucumerina sample. 272nm - Gallic acid RT -5.9

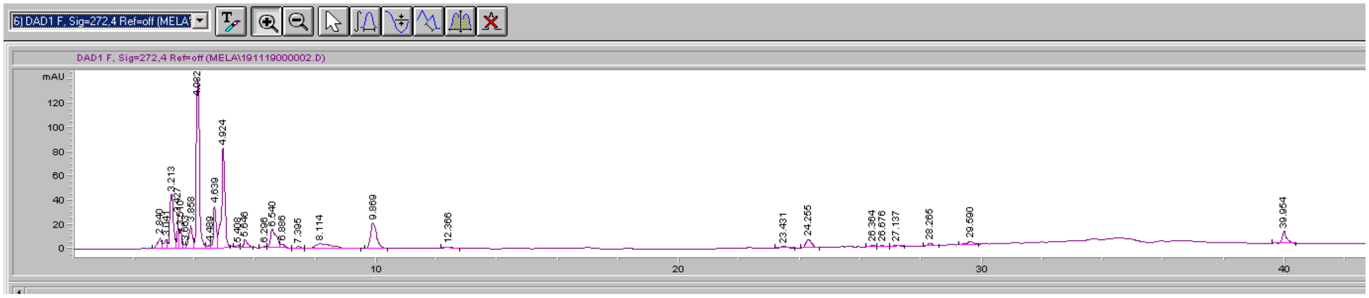

Figure S12. HPLC chromatogram of T. cucumerina sample. 280nm (+)-catechin hydrate (RT-17.6), (-)-epicatechin (RT-23.9)

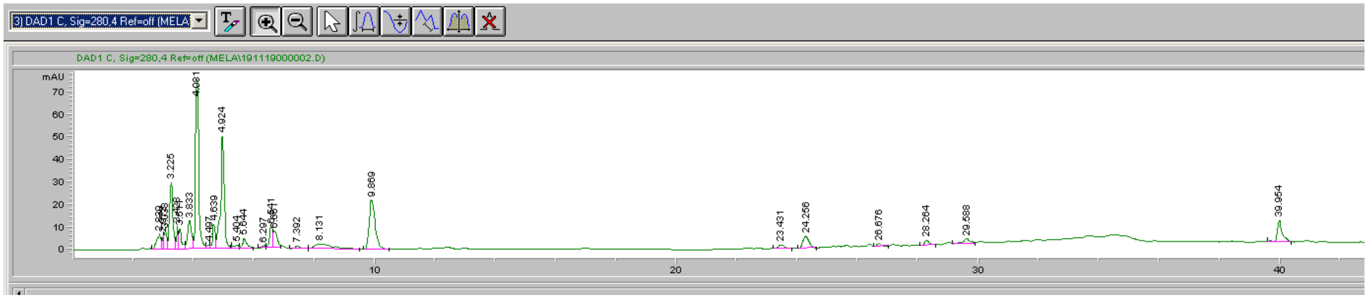

Figure S13. HPLC chromatogram of T. cucumerina sample. 325nm Neochlorogenic acid (RT-10.58), Chlorogenic acid (RT-22.3), Caffeic acid (RT-22.9), p-Coumaric acid (RT- 28.9) and trans-ferulic acid (RT-30.5)

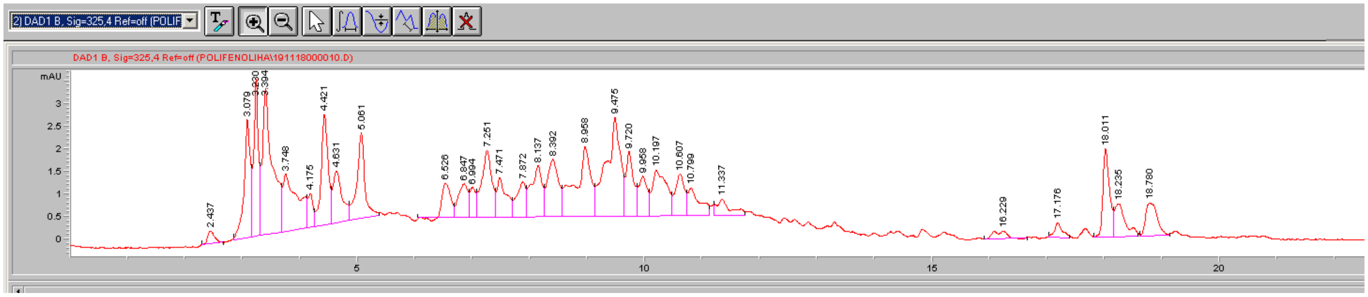

Figure S14. HPLC chromatogram of T. cucumerina sample. 365nm Kaempferol (RT-37.8) and Quercetin (RT- 35.8)

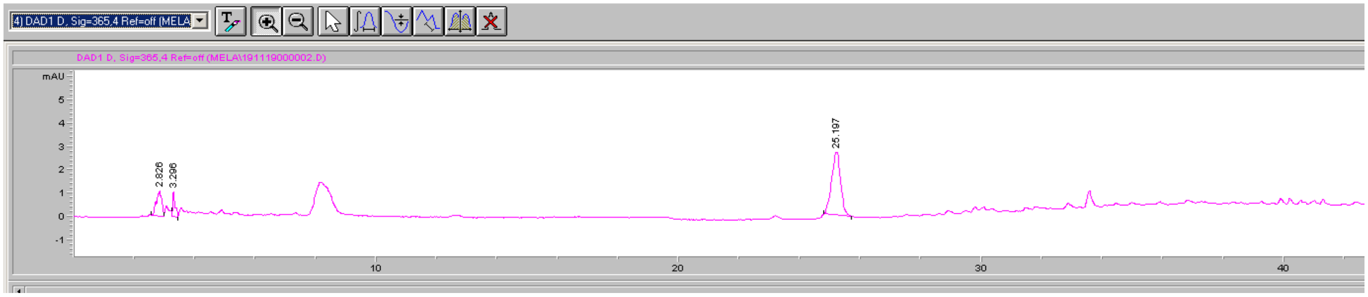

molecules

MDPI

Figure S15. HPLC chromatogram of *B. hispida* sample. 210nm Ursolic acid (RT-45.9) and Oleanolic acids (RT-45.8)

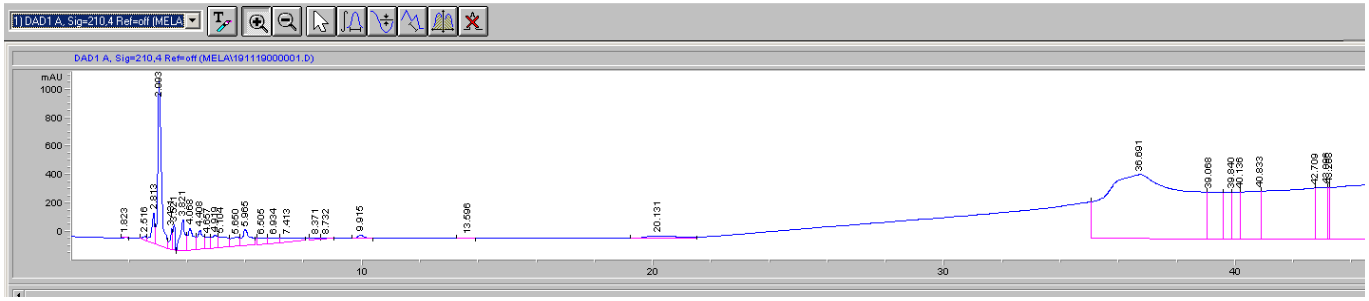

Figure S16. HPLC chromatogram of *B. hispida* sample. 230nm Procyanidin A2 (RT-24.3) and Procyanidin B2 (RT-29.9)

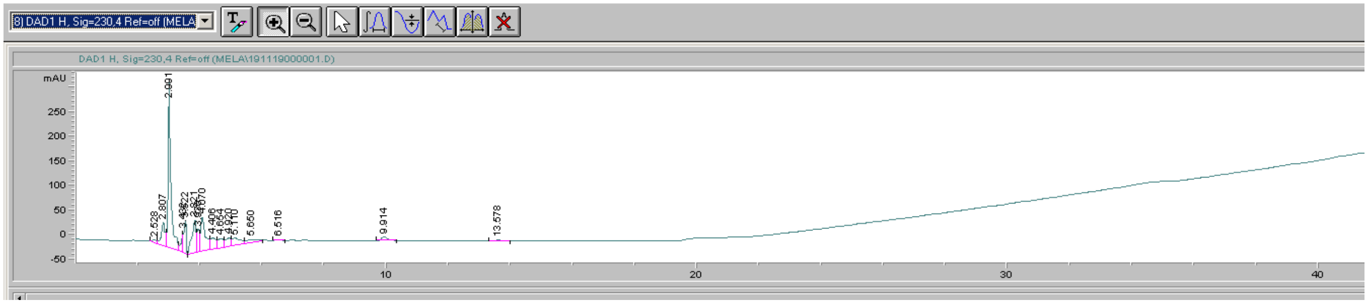

Figure S17. HPLC chromatogram of *B. hispida* sample. 265nm Rutin (RT-31.47), Quercetin-3-D-galactoside (RT-32) and Kaempferol-3-glucoside (RT-33.6)

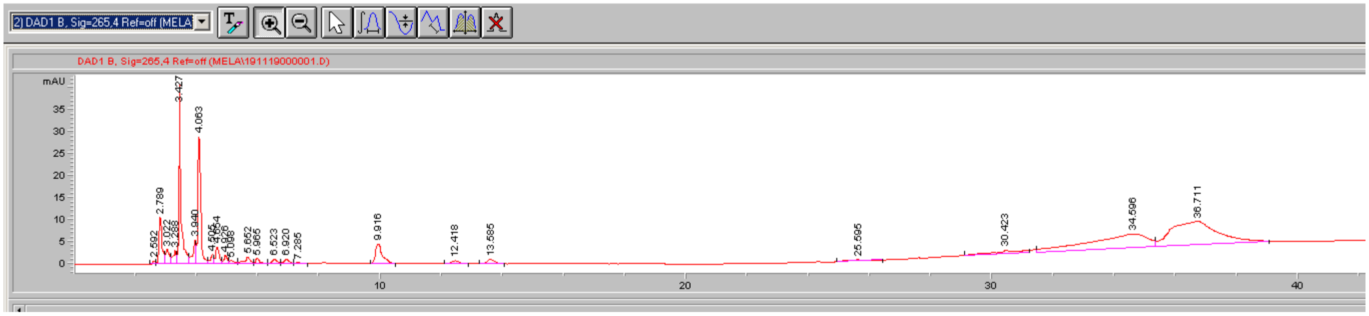

Figure S18. HPLC chromatogram of *B. hispida* sample. 272nm - Gallic acid RT -5.9

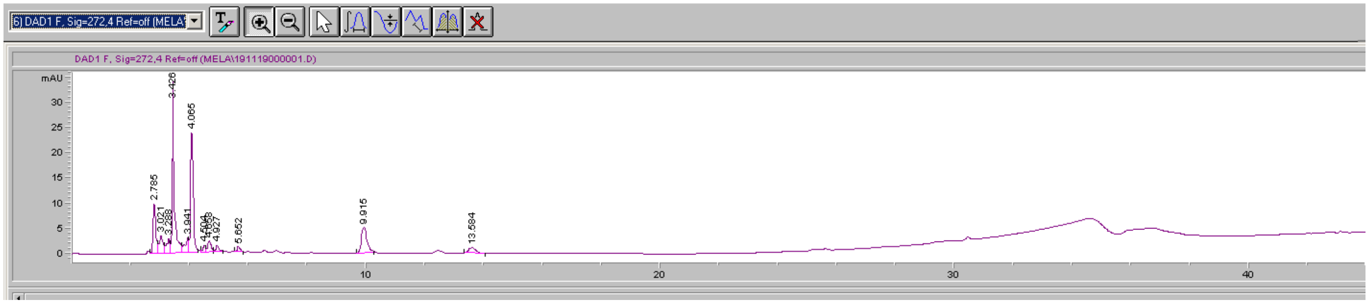

Figure S19. HPLC chromatogram of *B. hispida* sample. 280nm (+)-catechin hydrate (RT-17.6), (-)-epicatechin (RT-23.9)

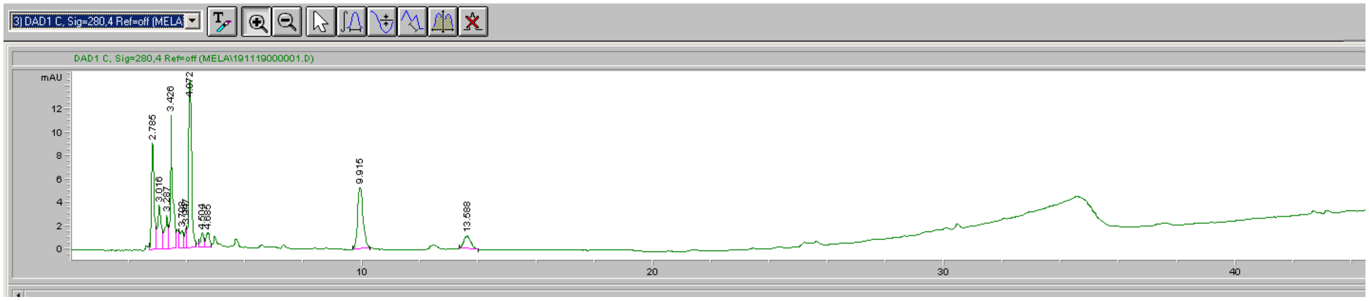

Figure S20. HPLC chromatogram of *B. hispida* sample. 325nm Neochlorogenic acid (RT-10.58), Chlorogenic acid (RT-22.3), Caffeic acid (RT-22.9), p-Coumaric acid (RT- 28.9) and trans-ferulic acid (RT-30.5)

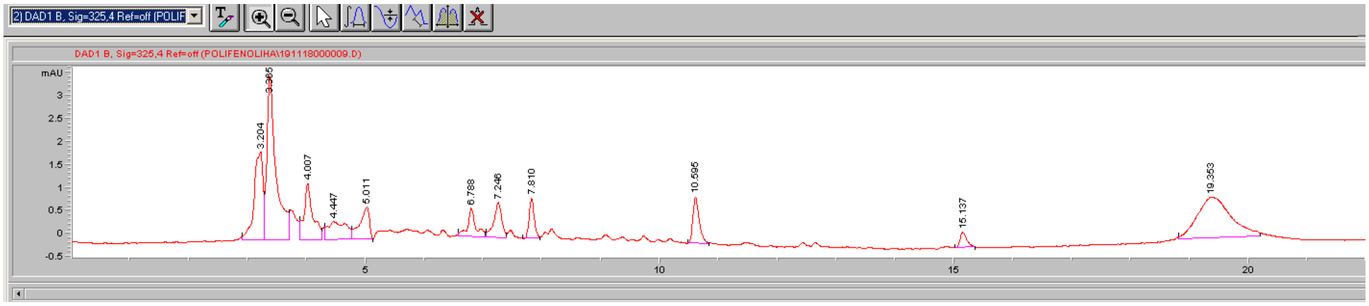

Figure S21. HPLC chromatogram of *B. hispida* sample. 365nm Kaempferol (RT-37.8) and Quercetin (RT-35.8)

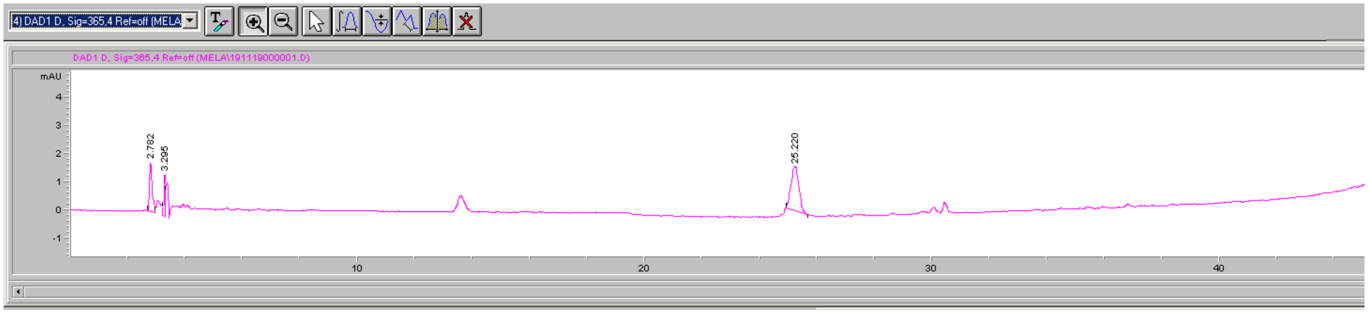

Figure S22. HPLC chromatogram of *C. metuliferus* sample. 210nm Ursolic acid (RT-45.9) and Oleanolic acids (RT-45.8)

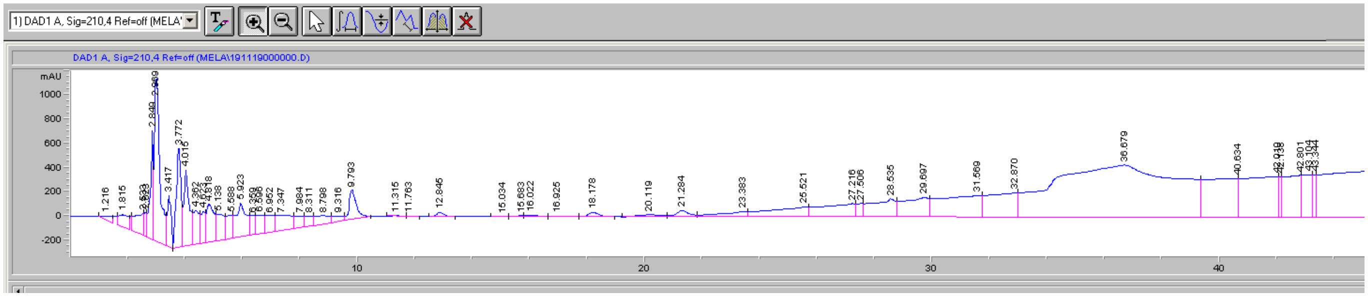

molecules

MDPI

Figure S23. HPLC chromatogram of *C. metuliferus* sample. 230nm Procyanidin A2 (RT-24.3) and Procyanidin B2 (RT-29.9)

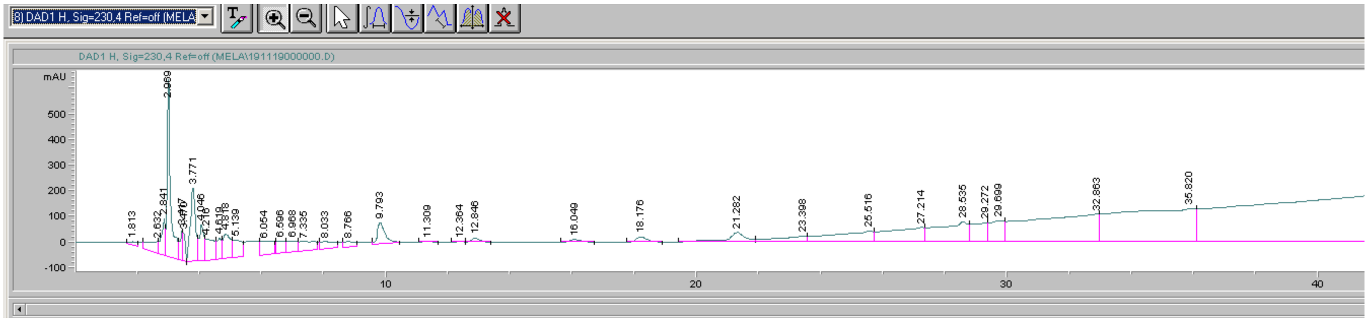

Figure S24. HPLC chromatogram of *C. metuliferus* sample. 265nm Rutin (RT-31.47), Quercetin-3-D-galactoside (RT-32) and Kaempferol-3-glucoside (RT-33.6)

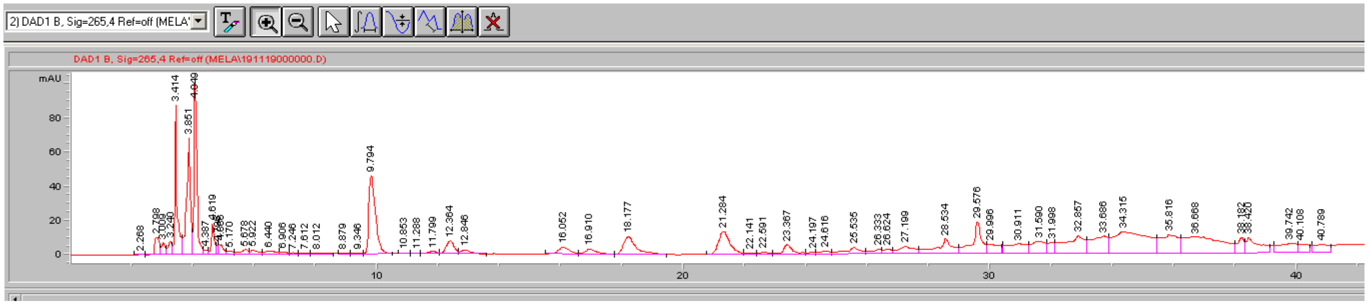

Figure S25. HPLC chromatogram of *C. metuliferus* sample. 272nm - Gallic acid RT -5.9

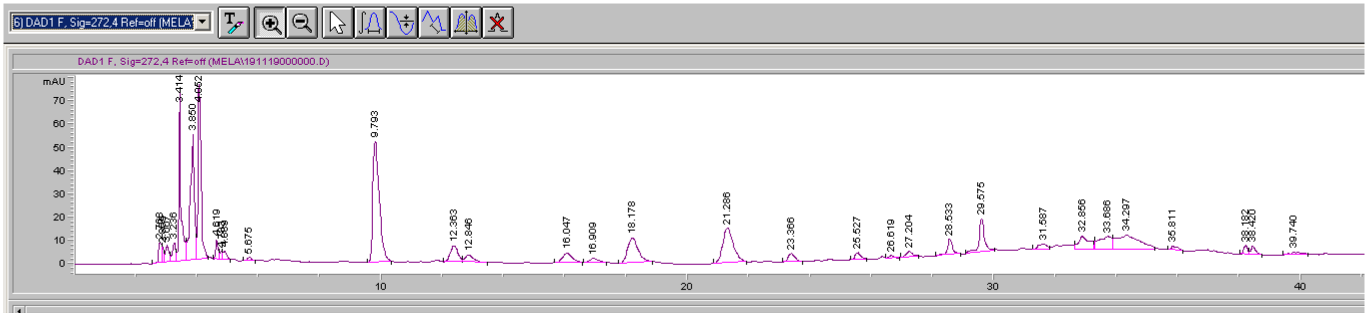

Figure S26. HPLC chromatogram of *C. metuliferus* sample. 280nm (+)-catechin hydrate (RT-17.6), (-)-epicatechin (RT-23.9)

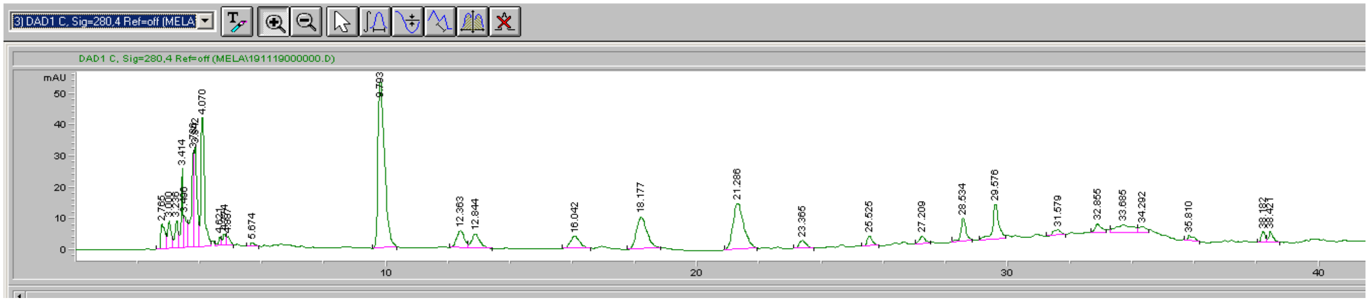

molecules

MDPI

Figure S27. HPLC chromatogram of *C. metuliferus* sample. 325nm Neochlorogenic acid (RT-10.58), Chlorogenic acid (RT-22.3), Caffeic acid (RT-22.9), p-Coumaric acid (RT- 28.9) and trans-ferulic acid (RT-30.5)

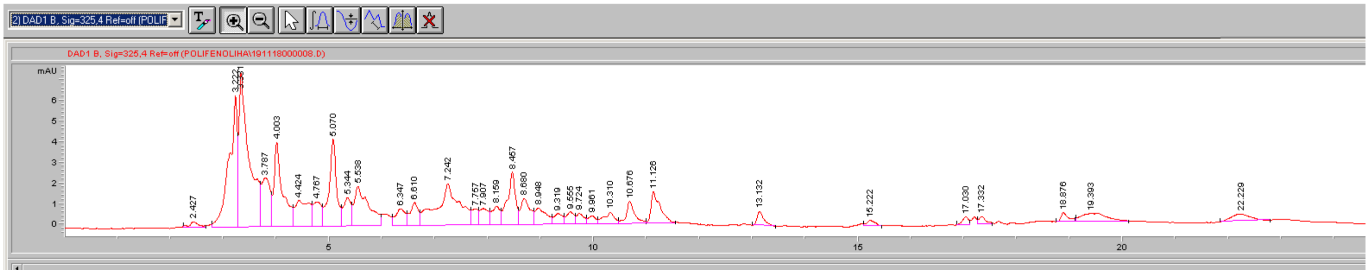

Figure S28. HPLC chromatogram of *C. metuliferus* sample. 365nm Kaempferol (RT-37.8) and Quercetin (RT- 35.8)

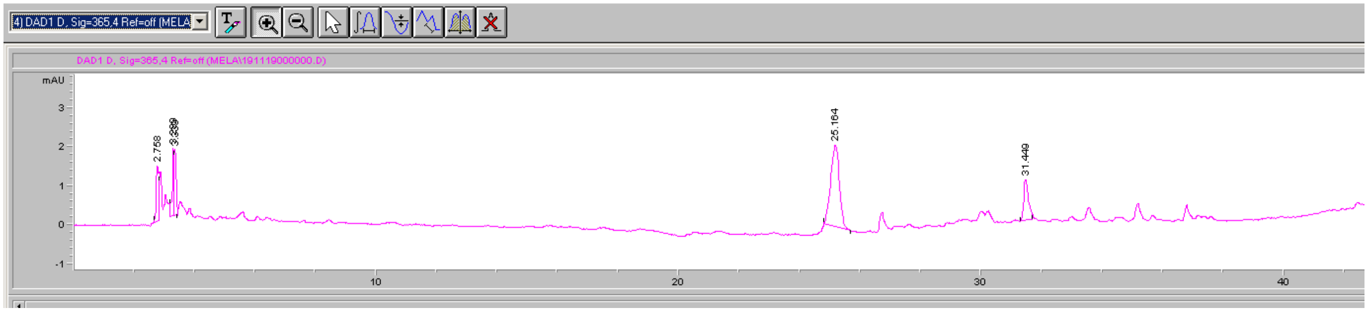

Supplement: Supplementary file 1 [file molecules-25-05468-s001.pdf]
